# Epigenetic Memory in Human Diseases: Acquired Chromatin States as Targetable Drivers of Disease Evolution

**DOI:** 10.3390/biom16101463

**Published:** 2026-10-08

**Authors:** Shahper Nazeer Khan, Danishuddin, Salamatu Tijjani Isah, Mohammed Haris Siddiqui, Aamir Ahmad, Jong-Joo Kim

**Affiliations:** 1 Integral Centre of Excellence for Interdisciplinary Research (ICEIR), Integral University, Lucknow 226026, India; 2 Department of Biosciences, Integral University, Lucknow 226026, India; salamatu@student.iul.ac.in; 3 Department of Biotechnology, Yeungnam University, 280 Daehak-ro, Gyeongsan 38541, Republic of Korea; danish23@yu.ac.kr; 4 Department of Bioengineering, Integral University, Lucknow 226026, India; 5 Hamad Medical Corporation, Doha 3050, Qatar

**Keywords:** epigenetic memory, clinical heterogeneity, single-cell epigenomics, therapy resistance, precision medicine

## Abstract

Clinical heterogeneity remains a major challenge across human diseases, where patients with similar genetic backgrounds frequently exhibit striking differences in disease progression, therapeutic response and clinical outcome. Increasing evidence indicates that these differences cannot be explained by genetic variation alone, positioning epigenetic memory as a critical non-genetic determinant of disease behavior. Epigenetic memory refers to the stable yet reversible propagation of transcriptional states through mechanisms including DNA methylation, histone modifications, chromatin accessibility, enhancer dynamics and higher-order chromatin organization. In this review, we synthesize emerging mechanistic and translational evidence demonstrating how epigenetic memory integrates environmental, metabolic, inflammatory, infectious and therapeutic cues into persistent cellular programs that drive inter-patient, intra-tumoral and temporal heterogeneity. We discuss its disease-specific manifestations across cancer, immunological, neurological, and metabolic disorders, with particular emphasis on cellular plasticity, stemness, immune evasion, drug-tolerant persister states and relapse. Furthermore, we highlight recent advances in single-cell and spatial epigenomics, liquid-biopsy-based epigenetic biomarkers, CRISPR-mediated epigenome editing and targeted chromatin therapies that collectively establish epigenetic memory as both a measurable biomarker and a therapeutically actionable vulnerability. By framing epigenetic memory as a dynamic interface between genome and environment, this review provides a unified conceptual framework for understanding disease evolution and advancing precision epigenetic medicine.

## 1. Introduction

**Figure 1:**
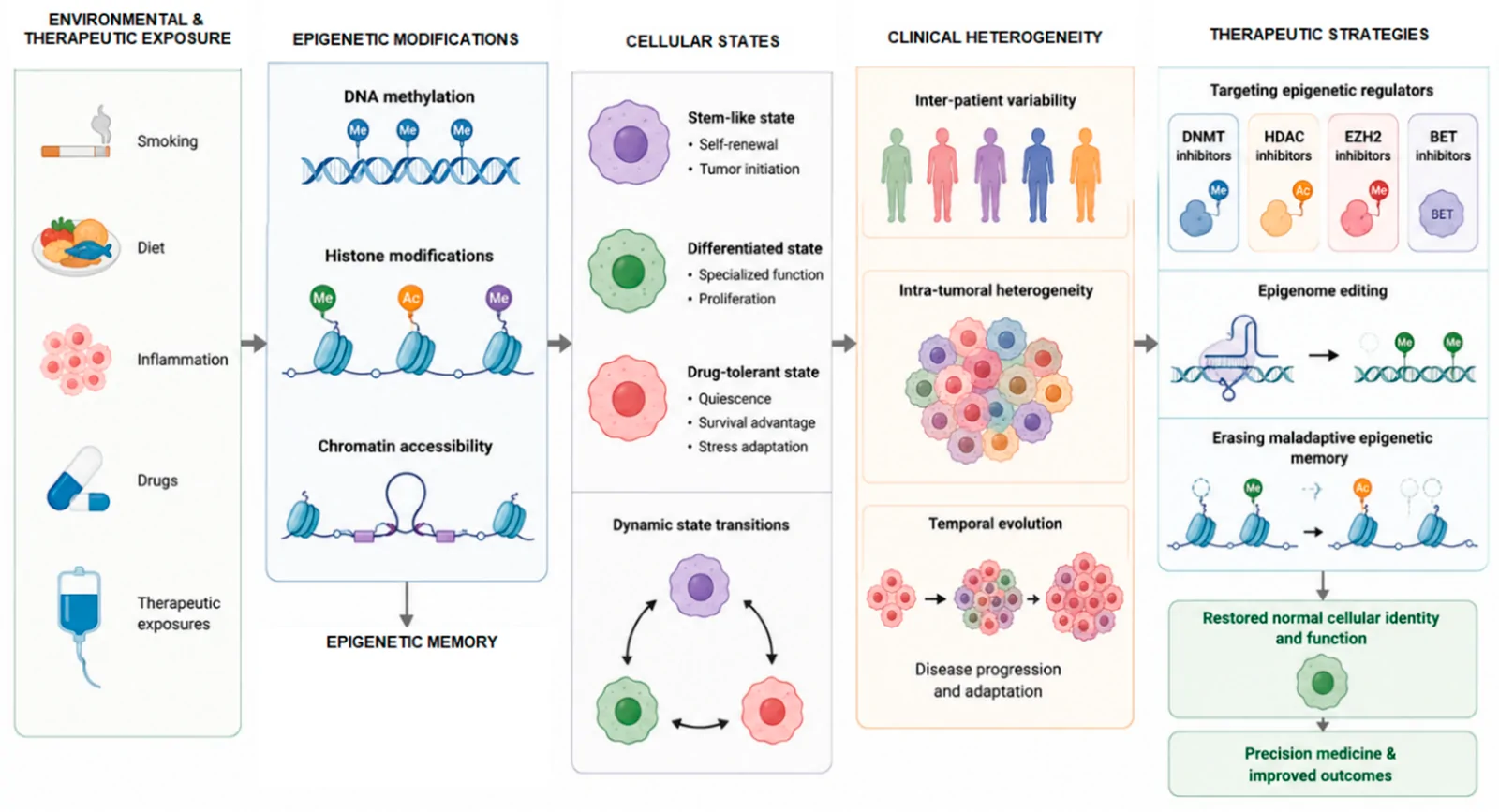
Conceptual Framework of Epigenetic Memory. Environmental and therapeutic exposures (e.g., smoking, diet, inflammation, drugs) induce stable epigenetic modifications, including DNA methylation and histone marks, that encode a persistent “memory” of prior stimuli. This epigenetic memory shapes cellular states promoting stem-like, differentiated, or drug-tolerant phenotypes—and drives dynamic state transitions. These processes collectively contribute to clinical heterogeneity, including inter-patient variability, intra-tumoral diversity, and temporal disease evolution. Therapeutic strategies targeting epigenetic regulators and epigenome editing aim to erase maladaptive epigenetic memory and restore normal cellular identity, enabling precision medicine.

Epigenetic memory refers to the ability of cells to maintain and transmit gene expression programs across cell divisions, or over prolonged periods in post-mitotic cells, without altering the underlying DNA sequence. This phenomenon is mediated through coordinated chromatin-based mechanisms, including DNA methylation, post-translational histone modifications, nucleosome positioning, chromatin accessibility, and higher-order genome architecture, which collectively stabilize cellular identity while retaining the potential for epigenetic reprogramming under appropriate conditions [1,2]. Environmental, metabolic, inflammatory, infectious, developmental, and therapeutic stimuli can induce persistent chromatin alterations that remain after the initiating stimulus has resolved, thereby encoding a molecular record of prior cellular experiences and shaping long-term cellular behaviour (Figure 1).

However, not all epigenetic alterations constitute bona fide epigenetic memory. In this review, we define epigenetic memory as a stable cellular phenotype or transcriptional program maintained through self-propagating epigenetic mechanisms after withdrawal of the initiating stimulus. Operationally, epigenetic memory is characterized by three defining features: (i) persistence after removal of the initiating stimulus; (ii) stable maintenance through one or more rounds of mitotic cell division or long-term persistence in post-mitotic cells; and (iii) experimental evidence demonstrating that DNA methylation, histone modifications, chromatin organization, or related chromatin-based mechanisms causally maintain the phenotype independent of alterations in DNA sequence. Here, we specifically focus on acquired epigenetic memory arising from environmental, metabolic, inflammatory, infectious, or therapeutic exposures and its contribution to pathological cellular states, disease progression, and clinical heterogeneity. This distinguishes the pathological context considered here from physiological epigenetic memory associated with embryonic development, lineage specification, and maintenance of differentiated cell identity. Persistent chromatin states are sustained through coordinated feedback involving DNA methyltransferases, histone-modifying enzymes, chromatin reader proteins, mitotic bookmarking, and higher-order genome organization, thereby reinforcing stable transcriptional programs and cellular phenotypes [1,2,3,4,5].

A fundamental challenge in modern medicine is the frequent discordance between genotype and phenotype, whereby individuals harboring similar genetic alterations often exhibit markedly different disease susceptibility, progression, and therapeutic responses. Such variability is particularly evident in cancer, autoimmune disorders, metabolic diseases, and neurodegenerative conditions, where genetic variation alone cannot fully account for clinical outcomes [6]. Increasing evidence indicates that acquired pathological epigenetic memory provides an additional layer of biological information that integrates previous environmental and cellular exposures into persistent chromatin states, thereby contributing to disease initiation, progression, therapeutic adaptation, and clinical heterogeneity. At the inter-patient level, persistent epigenetic variation contributes to differences in disease susceptibility, progression, and therapeutic response [7,8]. Within individual tumours, stable yet reversible epigenetic programs generate functionally distinct cellular subpopulations that differ in proliferative capacity, differentiation state, and drug sensitivity despite sharing an identical genetic background [9]. Over time, these persistent chromatin states facilitate dynamic transitions between cellular phenotypes during disease progression and in response to therapeutic intervention, thereby promoting tumour evolution, relapse, and acquired drug resistance [10]. Collectively, these observations establish epigenetic memory not merely as a molecular record of prior cellular experiences but as a mechanistic driver of stable transcriptional programs that sustain cellular plasticity and underlie clinical heterogeneity across human diseases. Unlike genetic mutations, which permanently alter the DNA sequence, epigenetic memory remains intrinsically reversible, making it an attractive therapeutic target [11,12]. Consequently, understanding how persistent chromatin states are established, maintained, and erased has important implications for disease prevention, biomarker discovery, precision medicine, and epigenetic reprogramming. While our previous reviews focused on epi-liquidomics and AI-driven precision medicine [13,14], this review aims to provide a mechanistic and translational framework for understanding acquired epigenetic memory as a persistent, reversible determinant of disease heterogeneity, progression, therapeutic response and relapse.

## 2. Mechanistic Basis of Epigenetic Memory

Epigenetic memory arises from interdependent molecular systems that preserve gene regulatory states through cell division while allowing context-dependent remodeling. Rather than being encoded by a single modification, it reflects the coordinated action of DNA methylation, histone modification networks, and chromatin accessibility, all embedded within higher-order regulatory dynamics. These layers collectively enable cells to maintain transcriptional programs despite the disruptive processes of DNA replication and chromatin turnover.

### 2.1. DNA Methylation Maintenance

DNA methylation at CpG dinucleotides provides a stable and heritable mark associated with long-term transcriptional repression. Its maintenance during replication is ensured by the DNMT1–UHRF1 axis, which recognizes hemi-methylated DNA and restores symmetric methylation patterns on daughter strands. UHRF1 binds hemi-methylated CpGs and coordinates histone ubiquitination, facilitating DNMT1 recruitment and activity at replication forks [15,16]. This coupling of DNA and histone signals enhances maintenance fidelity and ensures propagation of methylation patterns across cell divisions. Despite its stability, DNA methylation is dynamically regulated. Passive demethylation occurs through replication-dependent dilution when DNMT1 activity is reduced, whereas active demethylation is mediated by TET enzymes that oxidize 5-methylcytosine, leading to its replacement via base excision repair pathways. These opposing processes allow selective erasure or reinforcement of methylation states, enabling transitions between stable and flexible transcriptional programs [17,18]. Dysregulation of this balance, particularly aberrant maintenance or loss of methylation, is a hallmark of several diseases, including cancer, where global hypomethylation coexists with focal hypermethylation of tumor suppressor genes. Structural and biochemical studies further show that UHRF1-mediated ubiquitination of histone H3 provides a critical signal for DNMT1 recruitment, linking DNA methylation to chromatin context [19].

### 2.2. Histone Modification Networks

Histone modifications constitute a versatile and dynamic layer of epigenetic regulation, capable of encoding both stable and reversible transcriptional states. Central to this system is the antagonism between Polycomb group (PcG) and Trithorax group (TrxG) proteins, which maintain transcriptionally repressed and active chromatin, respectively. Polycomb repressive complex 2 (PRC2) deposits H3K27me3 marks associated with gene silencing, while Trithorax complexes catalyze H3K4 methylation linked to transcriptional activation [20]. A defining feature of epigenetic memory is the presence of bivalent chromatin domains, particularly in stem and progenitor cells. These domains harbor both activating (H3K4me3) and repressive (H3K27me3) marks, maintaining genes in a poised configuration that allows rapid activation or stable repression upon differentiation. Such states provide flexibility while preserving lineage commitment. Histone-based memory is further stabilized through read–write feedback loops, in which reader proteins recognize specific histone marks and recruit or cooperate with chromatin-modifying enzymes to reinforce existing chromatin states following replication. Examples include HP1α (CBX5), which recognizes H3K9 methylation; MPP8 (MPHOSPH8), which recognizes H3K9me3 and cooperates with the H3K9 methyltransferase SETDB1; and CBX7, which recognizes H3K27me3 and contributes to Polycomb-associated chromatin regulation. These reader–writer interactions contribute to the persistence and restoration of chromatin states following nucleosome displacement and DNA replication. Disruption of these feedback loops can lead to inappropriate stabilization of transcriptional programs, contributing to pathological states such as oncogenic silencing or aberrant activation.

### 2.3. Chromatin Accessibility and Enhancer Memory

Beyond covalent modifications, chromatin accessibility and enhancer dynamics play a critical role in sustaining transcriptional memory. Active enhancers are characterized by open chromatin, transcription factor binding, and histone acetylation marks such as H3K27ac. During mitosis, when global transcription is largely silenced, certain transcription factors remain associated with regulatory regions through mitotic bookmarking, enabling rapid reactivation of gene expression programs in daughter cells [21]. A subset of regulatory elements, termed super-enhancers, exhibit high densities of transcription factors and co-activators and are strongly associated with genes controlling cell identity. These regions demonstrate remarkable persistence and resistance to perturbation, reinforcing stable transcriptional states. In disease contexts, particularly cancer, aberrant activation or stabilization of super-enhancers can lock cells into pathogenic transcriptional programs, sustaining oncogenic signaling networks [22]. Emerging evidence suggests that enhancer landscapes themselves can act as repositories of epigenetic memory, integrating prior signaling events into long-term regulatory outputs.

### 2.4. Systems-Level Properties

**Figure 2:**
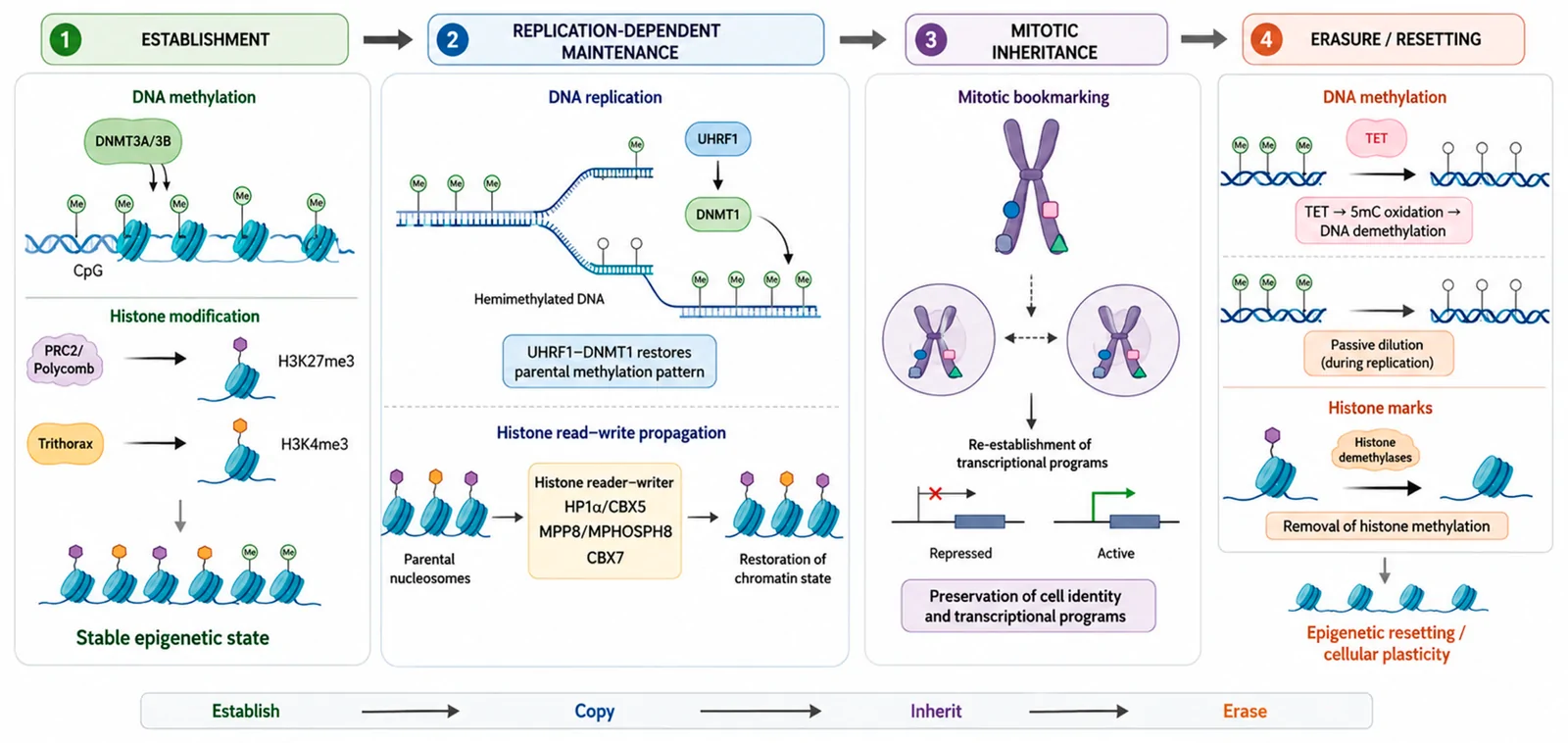
Mechanisms of epigenetic memory: establishment, maintenance, inheritance and erasure. (1) Establishment: DNMT3A/3B-mediated DNA methylation and histone modifications, including PRC2/Polycomb-associated H3K27me3 and Trithorax-associated H3K4me3, establish stable epigenetic states. (2) Replication-dependent maintenance: UHRF1 recognizes hemimethylated DNA and recruits DNMT1 to restore the parental methylation pattern, while histone reader–writer mechanisms involving HP1α/CBX5, MPP8/MPHOSPH8 and CBX7 contribute to chromatin-state propagation. (3) Mitotic inheritance: Mitotic bookmarking supports the preservation and re-establishment of transcriptional programs following cell division. (4) Erasure/resetting: TET-mediated 5mC oxidation and passive dilution contribute to DNA demethylation, while histone demethylases remove histone methylation, enabling epigenetic resetting and cellular plasticity.

At the systems level, epigenetic memory can emerge from nonlinear chromatin regulatory networks in which positive feedback stabilizes alternative transcriptional states. Mathematical and mechanistic studies have shown that feedback among chromatin-modifying processes can generate bistability, allowing active and repressed chromatin states to persist after withdrawal of an initiating stimulus. Hysteresis, in turn, describes the dependence of the current chromatin state on the system’s prior history and provides a mechanistic basis for persistent responses to transient stimuli [23,24]. In a recent study of *Saccharomyces cerevisiae* heterochromatin, bidirectional feedback between chromatin compaction and histone modification was shown to support bistable transcriptional states, highlighting the contribution of three-dimensional chromatin properties to epigenetic state stability [25]. Importantly, these findings derive primarily from theoretical models and experimental studies in yeast and should therefore be considered mechanistic principles rather than direct evidence of pathological epigenetic memory in human disease. Time-scale separation between the establishment, maintenance, and erasure of chromatin modifications can further influence the stability and duration of memory. Modeling studies indicate that when positive feedback and recruited modification processes operate faster than basal erasure, chromatin states become more stable and resistant to perturbation [23]. At the level of DNA replication, parental histone recycling and restoration of chromatin modifications provide additional mechanisms for maintaining epigenetic states. Recent experimental studies have demonstrated that replication-associated histone chaperones, including Mcm2 and Mrc1/CLASPIN, facilitate parental H3-H4 recycling and contribute to faithful transmission of heterochromatic states [26,27]. More broadly, interactions among DNA methylation, histone modifications, chromatin accessibility, and higher-order genome organization create multiple layers of feedback that can stabilize or destabilize transcriptional states. Together, these mechanisms illustrate a balance between chromatin stability and plasticity, allowing cellular states to remain sufficiently stable while retaining the capacity for regulated transitions in response to environmental or pathological cues (Figure 2).

## 3. Epigenetic Memory as a Driver of Clinical Heterogeneity

Acquired epigenetic memory can contribute to differences in disease progression, therapeutic response, and relapse by preserving exposure-induced cellular states over time. This persistent epigenetic variation may therefore represent an important contributor to clinical heterogeneity beyond underlying genetic differences.

### 3.1. Inter-Patient Heterogeneity

Inter-patient variability can persist even among individuals with comparable genetic backgrounds or shared driver mutations, reflecting differences in epigenetic landscapes that contribute to phenotypic diversity and disease behaviour [28,29]. Large-scale epigenomic profiling of primary human cancers has demonstrated substantial variation in chromatin accessibility and regulatory states across tumour types and patients [29]. Environmental and lifestyle exposures, including inflammation, metabolic stress, hypoxia, and toxicants, can further shape epigenetic states and contribute to persistent alterations in cellular function [5]. Importantly, persistent inflammatory signaling alone should not be considered evidence of epigenetic memory. In this review, inflammatory remodeling is considered acquired epigenetic memory only when an experimentally demonstrated chromatin state or transcriptional phenotype persists after reduction or removal of the initiating inflammatory stimulus and subsequently influences cellular responses. In immune disorders, inflammatory and infectious exposures can induce durable epigenetic reprogramming in innate immune cells, with trained immunity providing an experimentally supported example of persistent functional adaptation after the initiating stimulus [4]. Similarly, diabetes provides an example of metabolic memory, in which prior hyperglycaemic exposure is associated with persistent epigenetic alterations that contribute to vascular and inflammatory complications despite subsequent glycaemic control [30]. Together, these findings illustrate how acquired epigenetic states can integrate environmental and metabolic exposures into persistent cellular phenotypes, contributing to patient-specific disease trajectories beyond information encoded by the genome.

### 3.2. Cellular Heterogeneity

Epigenetic memory contributes to cellular heterogeneity by stabilizing distinct transcriptional programs within genetically similar populations of cells. Such heterogeneity arises from differences in chromatin accessibility, DNA methylation, histone modifications, enhancer activity, and transcription-factor networks, allowing individual cells to maintain distinct functional states despite sharing a similar genetic background [31]. Recent advances in single-cell epigenomic profiling further demonstrate that epigenomic heterogeneity and plasticity can generate a reservoir of distinct cellular states and contribute to disease evolution [32]. Importantly, epigenetic memory provides a mechanism through which cells can retain aspects of their previous state while remaining capable of transitioning toward alternative phenotypes. This creates a continuum of cellular states rather than discrete and irreversible cell identities. Chromatin remodeling and enhancer reprogramming can therefore establish transcriptional states that persist after the original stimulus has diminished, enabling cells to respond differently to subsequent environmental challenges. At the tissue level, such epigenetically maintained heterogeneity can influence cellular differentiation, functional specialization, and responses to changes in the microenvironment. Consequently, epigenetic memory acts as an important layer of phenotypic regulation that allows genetically similar cells to exhibit different behaviors and to dynamically adapt to changing conditions [32]. This non-genetic source of heterogeneity can complement genetic variation and contribute to temporal and spatial differences in disease progression and treatment response.

### 3.3. Therapy-Induced Memory

Therapeutic interventions can function as potent environmental stimuli that induce persistent epigenetic changes in exposed cells. Drug treatment, radiation, immune stimulation, and other therapeutic stresses can alter chromatin accessibility, DNA methylation, histone modifications, enhancer activity, and transcriptional networks. When such changes persist beyond the initial stimulus, they can establish a form of therapy-induced epigenetic memory that influences subsequent cellular responses [32]. A central feature of this process is the capacity of cells to retain information about previous exposure without requiring an immediate change in their underlying genetic sequence. Epigenetic remodeling can temporarily alter transcriptional programs and cellular functions, while subsequent restoration of the original chromatin state may permit phenotypic reversibility. Such transcriptional memory can involve persistent regulation of promoters and enhancers and may poise previously activated genes for more rapid reactivation following subsequent stimulation [33]. Thus, therapy-induced memory represents a dynamic interface between transient cellular adaptation and more durable phenotypic change. This principle also extends beyond malignant cells. In the immune system, for example, previous exposure to pathogens or inflammatory stimuli can induce persistent chromatin and transcriptional changes that alter responses to subsequent challenges. Trained immunity provides an established example in which innate immune cells undergo metabolic and epigenetic reprogramming following an initial stimulus, resulting in altered responsiveness upon re-exposure [4]. More broadly, such processes illustrate how cellular systems can encode aspects of their exposure history through epigenetic mechanisms. The persistence and reversibility of these epigenetic states may therefore influence how tissues respond to repeated environmental or therapeutic challenges. Depending on the cellular context, therapy-induced memory can facilitate adaptation, alter subsequent treatment responses, or provide a substrate from which more stable phenotypes may emerge [33]. Thus, epigenetic memory provides a conceptual framework for understanding how prior exposure can shape future cellular behavior and contribute to temporal heterogeneity in disease.

## 4. Disease-Specific Manifestations of Epigenetic Memory

**Figure 3:**
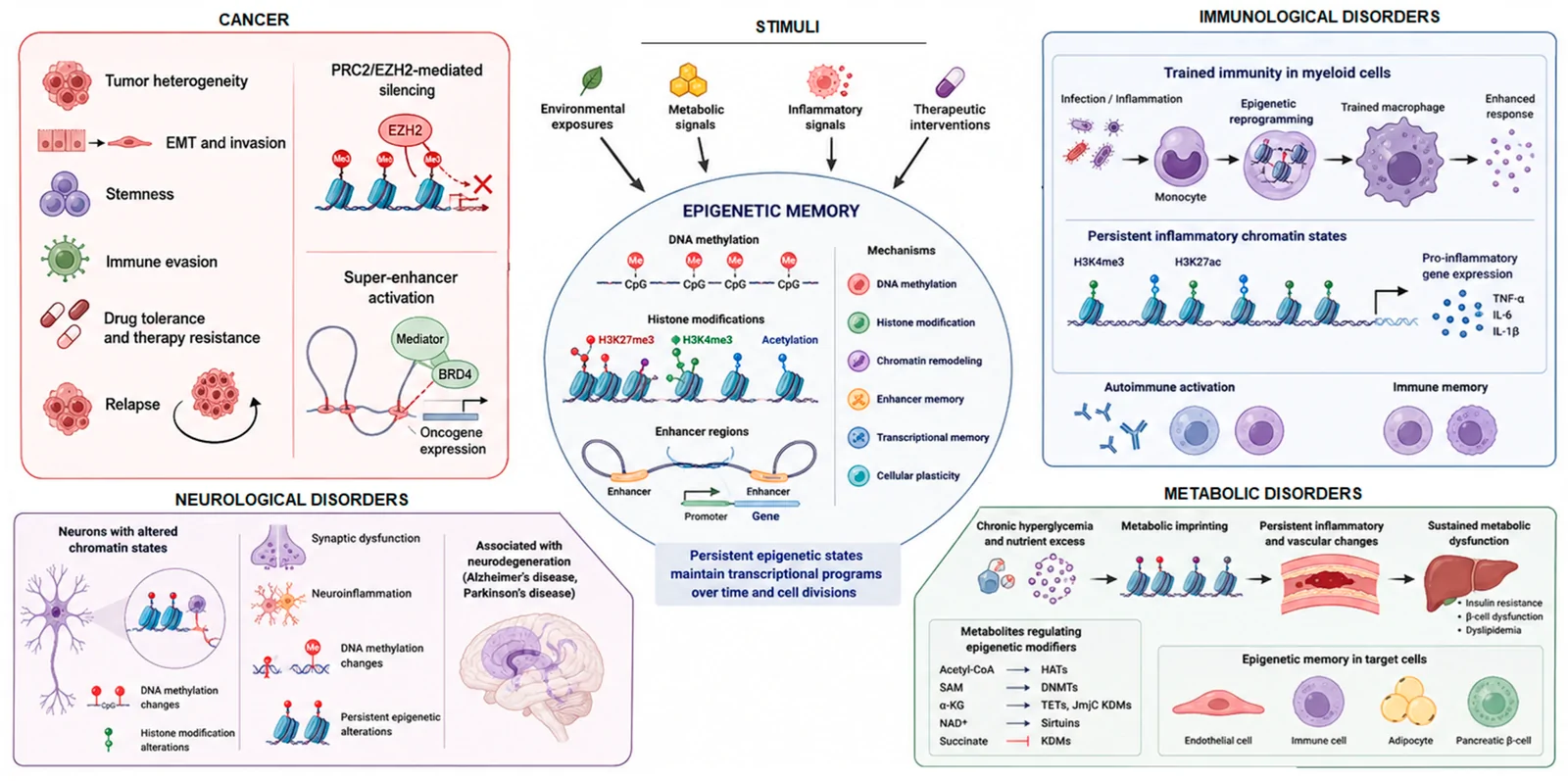
Epigenetic Memory Across Human Diseases. Environmental, metabolic, inflammatory, and therapeutic stimuli induce persistent yet reversible epigenetic alterations through DNA methylation, histone modifications, chromatin remodeling, enhancer memory, and altered chromatin accessibility, collectively establishing epigenetic memory. These stable chromatin states regulate transcriptional programs and cellular plasticity, contributing to disease progression and heterogeneity. In cancer, epigenetic memory drives tumor heterogeneity, EMT, stemness, immune evasion, drug tolerance, therapy resistance, and relapse via mechanisms including PRC2/EZH2-mediated silencing and super-enhancer activation. In immunological disorders, persistent inflammatory chromatin states promote trained immunity, autoimmune activation, and chronic inflammation. In neurological disorders, stable epigenetic alterations contribute to neuroinflammation, synaptic dysfunction, and neurodegeneration in Alzheimer’s and Parkinson’s disease. In metabolic diseases, hyperglycemic and metabolic memory sustain inflammatory and vascular dysfunction through metabolite-driven chromatin regulation.

Epigenetic memory manifests across disease contexts through context-dependent stabilization of transcriptional programs, enabling persistence of pathological states independent of ongoing stimuli. The diverse roles of epigenetic memory across cancer, immunological, neurological, and metabolic diseases are summarized in Figure 3. While the core mechanisms are conserved, their phenotypic consequences diverge across systems, reflecting disease-specific regulatory constraints and environmental inputs.

### 4.1. Oncology

In cancer, acquired epigenetic states can contribute to tumor heterogeneity, EMT-associated plasticity, stem-like phenotypes, immune evasion, drug tolerance, therapy resistance, and relapse when these states persist following an initiating oncogenic or therapeutic stimulus [34,35]. However, these phenotypes should not be considered synonymous with epigenetic memory. Rather, the relevant memory phenomenon is the persistence of an acquired chromatin and transcriptional state beyond the initiating stimulus, with a demonstrable influence on subsequent cellular behaviour. Epithelial-to-mesenchymal transition (EMT) is a particularly important example of cancer cell plasticity. EMT can be induced by signals including TGF-β, inflammatory cytokines, hypoxia, and oncogenic signaling and can occur through a spectrum of intermediate states rather than as an irreversible transition. Epigenetic remodeling can stabilize or bias EMT-associated transcriptional programs; however, EMT itself should not be classified as epigenetic memory unless the associated chromatin and transcriptional state persists after attenuation or withdrawal of the initiating signal and continues to influence subsequent cell-state transitions [36,37]. Similarly, cancer stem-like and progenitor-like phenotypes should not be equated with epigenetic memory. These cellular states can represent physiological or reversible states of cellular plasticity. In the context of acquired epigenetic memory, the relevant phenomenon is the persistence of an aberrantly acquired chromatin state that stabilizes or biases a stem-like transcriptional program following an initiating oncogenic, environmental, or therapeutic stimulus. Such persistent states may contribute to intratumoral heterogeneity and facilitate adaptation to changing microenvironments. Under therapeutic pressure, cancer cells can also enter drug-tolerant persister (DTP) states characterized by reversible transcriptional and chromatin alterations. Because DTP states can disappear following drug withdrawal, DTP formation alone should not be considered evidence of epigenetic memory [9]. Instead, DTP states are relevant to this review when therapy-associated chromatin alterations persist beyond drug withdrawal or alter the response to subsequent treatment. Such persistent epigenetic changes may provide a mechanistic link between transient drug tolerance and more durable therapy resistance. Additional longitudinal and functional evidence is required to establish this transition from reversible plasticity to acquired epigenetic memory. Epigenetic remodeling can also contribute to immune evasion through suppression of antigen-presentation machinery and interferon-responsive pathways, thereby reducing tumor recognition and potentially limiting responses to immunotherapy [38,39]. When such therapy- or inflammation-associated chromatin states persist after the initiating stimulus and influence subsequent immune responses, they may represent an acquired epigenetic memory mechanism. Collectively, these findings indicate that acquired epigenetic memory does not encompass cancer cell plasticity per se but rather represents the persistence and functional consequences of previously induced chromatin states that can bias subsequent EMT-associated, stem-like, drug-tolerant, immune-evasive, or therapy-resistant phenotypes.

### 4.2. Immunology

In immune systems, epigenetic memory governs both trained immunity and immune tolerance, shaping long-term responsiveness. Trained immunity involves sustained chromatin remodeling in innate immune cells following exposure to microbial or inflammatory stimuli, leading to enhanced cytokine production upon re-stimulation. This process is mediated by persistent histone modifications (e.g., H3K4me3, H3K27ac) at promoters and enhancers of inflammatory genes [3]. Conversely, tolerogenic states are established through epigenetic silencing of pro-inflammatory pathways, preventing excessive immune activation. In disease settings, persistent inflammatory imprinting may contribute to pathological immune activation; however, it is considered acquired epigenetic memory only when the altered chromatin or transcriptional state persists beyond the initiating inflammatory exposure or alters responses to subsequent stimulation [40,41]. In infectious diseases, epigenetic memory also influences variability in host responses, including differential outcomes in tuberculosis and viral infections [42].

### 4.3. Neurological Disorders

In the nervous system, epigenetic memory is particularly consequential due to the post-mitotic nature and longevity of neurons. Long-term chromatin remodeling, including DNA methylation changes and histone modifications, contributes to stable alterations in neuronal gene expression associated with neurodevelopment, synaptic plasticity, and neurodegeneration. In Alzheimer’s disease, widespread epigenomic alterations have been linked to dysregulation of immune and synaptic pathways, with single-cell studies revealing cell-type-specific chromatin changes [43,44]. Similarly, in Parkinson’s disease and other neurodegenerative disorders, persistent epigenetic dysregulation contributes to mitochondrial dysfunction, oxidative stress responses, and neuroinflammation [45]. Environmental exposures, including toxins and stress, further shape these epigenetic states, which can remain stable over extended periods. The durability of these modifications underscores the role of epigenetic memory in progressive and often irreversible disease phenotypes.

### 4.4. Metabolic Diseases

Metabolic diseases provide important examples of how environmental and metabolic exposures can become associated with persistent cellular states. In diabetes, transient hyperglycaemic exposure has been shown experimentally to induce persistent epigenetic alterations in vascular cells and animal models after restoration of normoglycaemia, providing mechanistic evidence for hyperglycaemic or metabolic memory [46,47]. Long-term clinical studies from the Diabetes Control and Complications Trial (DCCT)/Epidemiology of Diabetes Interventions and Complications (EDIC) cohort further demonstrated that the benefits of earlier intensive glycemic control persisted for many years after the original intervention, including sustained reductions in microvascular and cardiovascular complications [48,49]. These clinical observations support the long-term phenomenon of metabolic memory, while experimental studies provide evidence for a potential contribution of persistent epigenetic alterations to this process [47,50]. More broadly, metabolic state can influence chromatin regulation through metabolites such as acetyl-CoA, NAD^+^, and S-adenosylmethionine (SAM), which serve as substrates or cofactors for chromatin-modifying enzymes and thereby couple cellular metabolism to epigenetic regulation [51]. For example, changes in acetyl-CoA availability can influence histone acetylation, whereas changes in SAM availability can affect DNA and histone methylation. Conversely, epigenetic regulation of metabolic enzymes and pathways can alter cellular metabolism and thereby modify the intracellular availability of these metabolites. Thus, metabolic state can reshape chromatin, while chromatin regulation can subsequently influence metabolic state, establishing a reciprocal metabolism–epigenome feedback loop [51]. Such persistent metabolic-epigenetic interactions may contribute to differences in disease susceptibility and progression across metabolic and cardiovascular disorders. The diverse roles of epigenetic memory across cancer, immunological, neurological, and metabolic diseases are summarized in Figure 3.

## 5. Clinical Implications: Biomarkers and Patient Stratification

**Figure 4:**
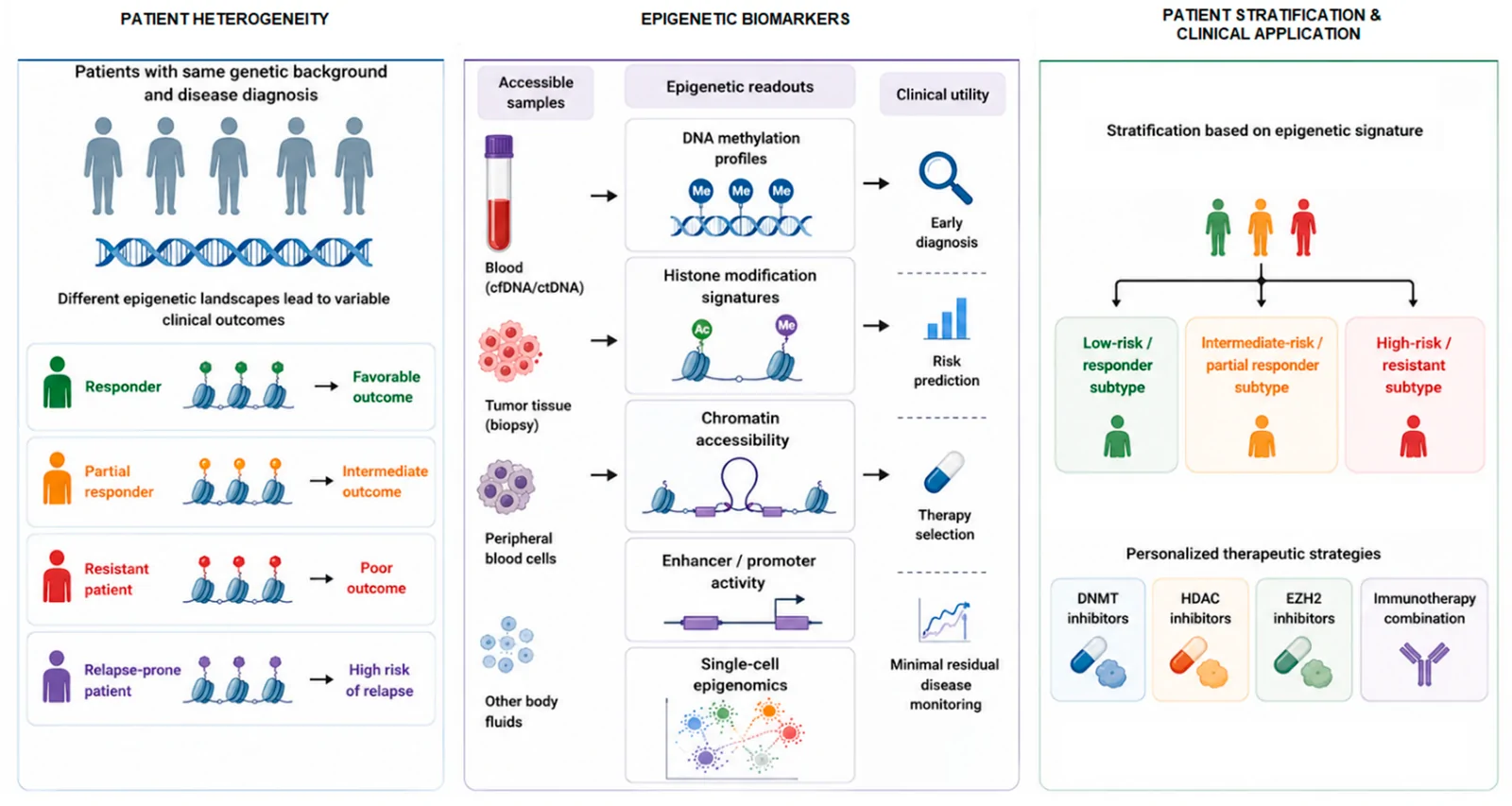
Clinical Implications of Epigenetic Memory: Biomarkers and Patient Stratification. Distinct epigenetic landscapes among patients with similar genetic backgrounds contribute to variable therapeutic responses and clinical outcomes. Epigenetic biomarkers, including DNA methylation, histone modifications, chromatin accessibility, enhancer activity, and single-cell epigenomic signatures, can be detected from blood, tumor tissue, and other clinical samples. These biomarkers support early diagnosis, risk prediction, therapy selection, and minimal residual disease monitoring. Patient stratification based on epigenetic signatures enables personalized therapeutic interventions, including DNMT, HDAC, and EZH2 inhibitors, ultimately improving treatment response and precision medicine outcomes.

The clinical relevance of epigenetic memory is increasingly reflected in biomarkers that capture disease-associated cellular states. Among epigenetic modalities, DNA methylation currently has the greatest clinical maturity, whereas chromatin accessibility and histone-mark profiling remain largely investigational. The principal epigenetic biomarker modalities, their clinical applications, and current levels of clinical maturity are summarized in Table 1, while their potential roles in diagnosis, risk prediction, therapy selection, and patient stratification are illustrated in Figure 4.

### 5.1. Epigenetic Biomarkers

DNA methylation represents the most clinically advanced class of epigenetic biomarkers. Methylation signatures can distinguish malignant from non-malignant tissues and can be detected in circulating cell-free DNA (cfDNA), enabling minimally invasive disease assessment. In a large validation study, a cfDNA methylation-based multi-cancer assay achieved 99.3% specificity and 67.3% sensitivity for stages I–III across a prespecified set of 12 cancer types, although sensitivity varied substantially by cancer type and disease stage [53,61]. Methylation profiles can also provide information on tissue of origin, with high classification accuracy demonstrated in multi-cancer studies [61]. Beyond DNA methylation, chromatin-accessibility profiling using ATAC-seq can delineate disease-associated regulatory landscapes and cellular states, although its translation into routine clinical practice remains investigational [62]. Similarly, profiling of histone modifications such as H3K27ac and H3K27me3 using low-input Cleavage Under Targets and Release Using Nuclease (CUT&RUN) or Cleavage Under Targets and Tagmentation (CUT&Tag) is emerging as a potentially useful approach for characterizing regulatory states, but requires further clinical validation [63]. Both methods use antibody-guided targeting of chromatin-associated proteins or histone modifications, followed by localized DNA cleavage or transposase-mediated tagging, respectively, enabling low-input genome-wide profiling with reduced background compared with conventional chromatin immunoprecipitation sequencing (ChIP-seq).

### 5.2. Clinical Applications

Epigenetic biomarkers have applications in diagnosis, prognosis, and therapy selection, although their clinical maturity varies by biomarker and disease. In colorectal cancer, circulating methylated SEPT9 has demonstrated clinically relevant diagnostic performance, with pooled sensitivity of approximately 69% and specificity of 92% across published studies [64]. However, performance varies according to assay design, disease stage, and study population [64]. DNA methylation profiling can also improve tumor classification and patient stratification. Methylation-based classifiers have demonstrated high accuracy for identifying tumor type and tissue of origin, including cancers of unknown primary [65]. In glioblastoma, MGMT promoter methylation is an established molecular biomarker associated with prognosis and response to alkylating-agent therapy [66]. Epigenetic biomarkers are also being investigated for treatment-response prediction and minimal residual disease (MRD) monitoring. Methylation patterns in circulating tumor DNA can provide a minimally invasive means of tracking tumor burden and treatment response; however, these applications remain under prospective clinical evaluation [7]. Similarly, epigenetic states associated with immune-related regulatory regions have been investigated as potential predictors of response to immune checkpoint blockade, but these approaches remain investigational [67].

### 5.3. Advantages over Genomics

Epigenetic biomarkers complement genomic alterations by capturing dynamic cellular and transcriptional states that may change during disease progression and treatment. Their potential reversibility allows longitudinal assessment of treatment response and emerging resistance, while cfDNA methylation enables minimally invasive monitoring [53,61]. Epigenetic profiles can also reflect environmental, inflammatory, and metabolic influences, providing information that may not be captured by genomic alterations alone [7]. Therapy-associated epigenetic remodeling has been documented across cancer treatment settings. For example, treatment with DNA methyltransferase inhibitors such as azacitidine or decitabine can reduce DNA methylation and reactivate epigenetically silenced genes, whereas histone deacetylase inhibitors alter histone acetylation and chromatin accessibility. Conversely, therapeutic pressure can select or induce alternative chromatin states associated with drug tolerance and resistance. These changes illustrate why longitudinal epigenomic profiling may provide information about treatment response and emerging resistance beyond static genomic measurements. Thus, integration of epigenomic and genomic information may provide a more comprehensive representation of disease heterogeneity and evolution.

### 5.4. Limitations

Despite their potential, several challenges limit the routine clinical implementation of epigenetic biomarkers. Pre-analytical variation, differences in sample processing, sequencing platforms, bioinformatic pipelines, and normalization procedures can affect reproducibility and cross-study comparability [68]. Cell-type specificity further complicates interpretation, particularly in liquid-biopsy applications where tumor-derived signals are mixed with cfDNA from non-malignant tissues [69]. Low tumor-derived cfDNA abundance in early-stage disease can also limit assay sensitivity, necessitating highly sensitive analytical methods and rigorous validation [70]. Moreover, many emerging epigenetic biomarkers have been evaluated predominantly in retrospective or case–control cohorts and require prospective validation and demonstration of clinical utility before widespread adoption. Therefore, while selected DNA methylation biomarkers have achieved clinical application, chromatin accessibility, histone-mark profiling, and epigenome-based longitudinal monitoring should currently be regarded as emerging rather than established clinical approaches. A further challenge is the quantitative definition and measurement of epigenetic memory at the cellular and genomic levels. Bulk epigenomic profiling provides an average signal across heterogeneous cell populations and therefore cannot readily determine whether a persistent epigenetic state is present in most cells or restricted to a small subpopulation. Single-cell DNA methylation, chromatin-accessibility, and histone-mark profiling can help resolve epigenetic states at cellular resolution and estimate the fraction of cells carrying a particular state. However, demonstrating epigenetic memory requires longitudinal assessment or experimental removal of the initiating stimulus to establish persistence, rather than simply detecting an epigenetic difference at a single time point. At present, there is no universally accepted quantitative threshold for the proportion of cells or genomic loci that must retain an epigenetic alteration for it to be defined as clinically meaningful epigenetic memory. Establishing quantitative criteria, together with longitudinal and functional validation, therefore remains an important challenge for translating epigenetic memory into measurable clinical biomarkers.

## 6. Targeting Epigenetic Memory

The recognition of epigenetic memory as a driver of disease heterogeneity has catalyzed a shift toward therapeutic strategies that modulate chromatin states rather than static genetic lesions. Unlike genomic alterations, epigenetic marks are inherently reversible, positioning them as attractive targets for pharmacological reprogramming and precision intervention.

### 6.1. Epigenetic Drugs

The first generation of epigenetic therapies has focused primarily on DNA methyltransferase (DNMT) inhibitors and histone deacetylase (HDAC) inhibitors, which broadly alter chromatin states and transcriptional programs. DNMT inhibitors such as azacitidine and decitabine are established therapies for myelodysplastic syndromes (MDS) and acute myeloid leukemia (AML). Their therapeutic effects involve DNA hypomethylation and reactivation of epigenetically silenced genes, although their biological consequences extend beyond simple global demethylation and are highly context-dependent. Clinical studies have reported response or clinical-improvement rates generally ranging from approximately 30–50% across different MDS populations, treatment schedules, and response criteria, with azacitidine demonstrating a survival advantage in higher-risk MDS [11,54]. However, responses are frequently incomplete or transient, highlighting the limitations of broadly acting epigenetic agents and the complexity of methylation-dependent regulation. HDAC inhibitors, including vorinostat and romidepsin, increase histone acetylation and can alter chromatin accessibility and transcriptional activity. These agents have demonstrated clinically meaningful activity in T-cell lymphomas, with reported overall response rates of approximately 30% for vorinostat and 34% for romidepsin in cutaneous T-cell lymphoma [71]. Nevertheless, their broader therapeutic application has been constrained by treatment-related toxicities, modest response rates, and limited durability in many disease settings. More recently, targeted epigenetic therapies have emerged that inhibit specific chromatin regulators, including enhancer of zeste homolog 2 (EZH2) and bromodomain and extraterminal (BET) proteins. The EZH2 inhibitor tazemetostat has demonstrated clinically meaningful activity in relapsed or refractory follicular lymphoma, particularly in tumors harboring activating EZH2 mutations [72]. Tazemetostat has also demonstrated activity in epithelioid sarcoma characterized by loss of INI1/SMARCB1, illustrating how biomarker-defined epigenetic dependencies can support more precise therapeutic strategies. BET inhibitors, which interfere with BET protein-dependent transcription at active regulatory elements and can suppress oncogenic programs including MYC-associated transcription, have shown promising preclinical activity but more limited and variable clinical efficacy. Adaptive and acquired resistance, together with dose-limiting toxicities, remain important barriers to their clinical development [73]. Collectively, these findings demonstrate that pharmacological perturbation of epigenetic states can produce therapeutic benefit while emphasizing the need for biomarker-guided target selection, greater specificity, and durable disease control.

### 6.2. Reprogramming Strategies

Unlike clinically established epigenetic drugs and investigational pharmacological approaches, CRISPR-dCas9-based epigenome editing remains a preclinical and experimental strategy. Current evidence is derived predominantly from cellular and animal models, with important challenges related to delivery, locus specificity, durability, off-target effects and safety that must be addressed before clinical translation. For example, dCas9-DNMT3A and dCas9-TET1-based systems can be used to induce targeted DNA methylation or demethylation, respectively, providing a framework for precise control of gene expression and the potential rewriting of disease-associated epigenetic states [74]. More broadly, epigenetic therapy requires a balance between chromatin stability and plasticity: treatment may seek either to disrupt stable, disease-driving epigenetic states or to exploit transient windows of cellular plasticity to increase therapeutic sensitivity and immune recognition. Resistance can emerge through adaptive chromatin reprogramming and compensatory transcriptional pathways, whereas broad-spectrum agents such as DNMT and HDAC inhibitors may produce systemic toxicities and unintended transcriptional effects. The context-dependent nature of epigenetic regulation further complicates patient selection. Consequently, temporal treatment strategies, predictive biomarkers, and increasingly integrated single-cell, spatial, and multi-omics approaches will be important for achieving more precise and durable epigenetic therapies [9,28,32,35].

## 7. Future Directions

The next phase of epigenetic memory research is being driven by technological convergence and scale, fundamentally reshaping how disease heterogeneity is decoded and clinically translated. Advances in single-cell and spatial epigenomics now enable simultaneous profiling of DNA methylation, chromatin accessibility, histone modifications, and 3D genome architecture at single-cell resolution within intact tissues. These approaches can resolve rare cellular populations (<1%) and reconstruct lineage trajectories, providing direct insight into how epigenetic memory is established and maintained in spatially defined microenvironments [75]. Recent computational approaches further demonstrate that spatial transcriptomics can be integrated with single-cell multi-omics to infer spatially resolved chromatin accessibility and gene-regulatory networks, providing a framework for examining epigenomic states within tissue microenvironments [76]. A recent report also introduces quantitative epigenetic heterogeneity metrics, linking intra-tumoral chromatin variability with clinical outcomes and therapy resistance [77]. Parallel developments in multi-omics integration are transitioning the field from descriptive to mechanistic biology. They further enable the simultaneous profiling of genome conformation, histone modifications, chromatin accessibility, and gene expression within individual cells, providing an integrated view of gene-regulatory landscapes and enhancer–promoter relationships [78]. Current large-scale consortia datasets routinely include **10^5^–10^6^ cells per study**, allowing robust modeling of rare but clinically relevant states and transitions [79]. A major frontier is the application of artificial intelligence (AI) and deep learning models to predict epigenetic state dynamics. Recent foundation-model approaches based on large-scale Hi-C datasets demonstrate the potential of AI to integrate three-dimensional genome architecture with epigenomic information and to extend chromatin-architecture analysis to single-cell and multi-omics datasets [80]. These models are increasingly capable of simulating cell state transitions and perturbation responses, offering a predictive framework for disease progression and therapeutic intervention. However, challenges remain in model interpretability, cross-cohort generalizability, and integration into clinical workflows [81]. Importantly, recent experimental evidence directly demonstrates that inflammatory exposure can generate persistent epigenetic memory with functional consequences. In a mouse model of colitis, inflammatory exposure produced durable epigenetic states in colonic stem cells that persisted for more than 100 days after disease resolution and were associated with subsequent tumor-promoting responses [82]. Collectively, these advances are converging toward precision epigenomics, where patient-specific chromatin landscapes guide diagnosis, prognosis, and therapy selection. Longitudinal epigenomic profiling combined with AI-driven prediction may enable real-time monitoring of disease evolution, supporting adaptive therapeutic strategies. A central unresolved question remains: Can acquired epigenetic memory be selectively erased without compromising normal cellular identity? Addressing this will require precise epigenome editing, deeper understanding of chromatin state hierarchies and rigorous clinical validation.

## 8. Conclusions

Epigenetic memory has emerged as a central determinant of disease heterogeneity, providing a mechanistic link between genotype, environment, and phenotype. By stabilizing transcriptional programs through coordinated regulation of DNA methylation, histone modifications, and chromatin accessibility, it drives persistent cellular states that influence disease initiation, progression, and therapeutic response. Unlike genetic alterations, epigenetic memory is inherently dynamic, reversible, and context-dependent, allowing it to capture prior environmental exposures, metabolic conditions, and treatment history. This makes it both measurable through emerging epigenomic technologies and amenable to therapeutic modulation. Advances in single-cell and spatial epigenomics, coupled with liquid biopsy approaches and epigenome editing tools, are accelerating its translation into clinical practice. These developments position epigenetic memory as a critical layer for patient stratification and precision medicine.

## Figures and Tables

**Table 1:** Disease-Specific Epigenetic Memory Mechanisms and Their Clinical and Therapeutic Implications.

Disease/State	Key Epigenetic Mechanisms	Quantitative Evidence/Facts	Functional Consequences	Clinical Heterogeneity	Therapeutic Targeting
Oncology	DNA methylation, Polycomb/EZH2, chromatin remodeling, super-enhancers	60% of pancreatic carcinomas (27/45) showed aberrant methylation at ≥1 locus in a primary tumor study [52]. Methylation-based cfDNA detection achieved 99.3% specificity and 67.3% sensitivity for stages I–III cancers in a prespecified 12-cancer set [53]. DNMT inhibitors produce variable responses across myeloid malignancies; for example, phase III azacitidine data reported 18% CR in one AML cohort, while other studies reported higher composite response rates [54].	EMT, stemness, immune evasion, adaptive survival	Inter- and intra-tumoral epigenetic heterogeneity; reversible persister states	DNMTi, HDACi, EZH2i, BETi; combinations with immunotherapy
Immunology	Histone marks, chromatin accessibility, metabolic–epigenetic regulation	In a primary human-monocyte model, β-glucan training produced increased cytokine responses after restimulation; the study reported a significant increase in cytokine production under specific training conditions [55].	Persistent inflammatory activation or tolerance	Variable trained immune responses	Epigenetic/metabolic modulation + immunotherapy
Neurological Disorders	DNA methylation, histone acetylation, chromatin remodeling	AD EWAS/meta-analysis identified 236 significant CpGs in prefrontal cortex, 95 in temporal gyrus, and 10 in entorhinal cortex associated with Braak stage [56]. A separate multi-region study identified 858 robust differentially methylated sites across four brain regions [57].	Synaptic dysfunction, altered neuronal regulation, neurodegeneration	Region- and disease-stage-dependent epigenetic heterogeneity	HDAC inhibitors; epigenome editing (emerging)
Metabolic Diseases	Histone modifications, DNA methylation, metabolite-driven regulation	Experimental studies demonstrate that transient hyperglycemia can produce persistent epigenetic and transcriptional changes after return to normoglycemia, supporting metabolic/hyperglycemic memory [46].	Sustained inflammation, endothelial dysfunction, vascular complications	Persistent complications despite glycemic normalization	Metabolic–epigenetic targeting; lifestyle interventions
Therapy-Induced States (Cancer)	Chromatin reprogramming, enhancer rewiring	Sharma et al. identified DTPs at approximately 0.3% of the starting PC9 population, with DTP frequencies of approximately 0.3–5% across tested cancer cell models; the state was reversible after drug withdrawal and associated with altered chromatin regulation [58].	Survival under therapy, quiescence	Adaptive resistance, persistence, relapse	Epigenetic priming; combination therapy
Aging & Environmental Exposure	Global hypomethylation, locus-specific methylation changes	A longitudinal twin study identified 2284 CpG sites showing DNA-methylation changes over 10 years, with >60% replicated in an independent sample [59]. DNA-methylation-based age prediction showed a correlation of 0.96 with chronological age and a median error of 3.6 years in the test dataset [60].	Altered gene regulation and cellular function	Inter-individual variation in biological aging	Preventive and age-modifying interventions

## Data Availability

No new data were created or analyzed in this review. Data sharing is not applicable to this article.
